# Microstructure and Tensile Properties of ECAPed Mg-9Al-1Si-1SiC Composites: The Influence of Initial Microstructures

**DOI:** 10.3390/ma11010136

**Published:** 2018-01-15

**Authors:** Shaoxiong Zhang, Ming Li, Hongxia Wang, Weili Cheng, Weiwei Lei, Yiming Liu, Wei Liang

**Affiliations:** 1 Shanxi Key Laboratory of Advanced Magnesium-Based Materials, College of Materials Science and Engineering, Taiyuan University of Technology, Taiyuan 030024, China; zhangshaoxiong0175@link.tyut.edu.cn (S.Z.); liming9131217@163.com (M.L.); lww1836@126.com (W.L.); liuym812@163.com (Y.L.); liangwei@TYUT.edu.cn (W.L.); 2 Southwest Technique and Engineering Research Institute, Chongqing 400039, China

**Keywords:** Mg-9Al-1Si-1SiC composite, homogenization treatment, ECAP, microstructure, mechanical properties

## Abstract

Mg-9Al-1Si-1SiC composites with various initial microstructures prior to equal channel angular pressing (ECAP) were obtained by different pre-treatments (without and with homogenization treatment), and the resultant grain size, second phase and tensile properties of ECAPed composites were reported. The ECAPed composite with homogenization treatment (HT) exhibited finer grain size, higher fraction of dynamically recrystallized (DRXed) grains, weaker texture intensity, as well as the presence of dynamic precipitated Mg_17_Al_12_ phase compared to that without HT. Besides, the morphology of pre-existing Mg_2_Si changed from massive-like to needle-like in the ECAPed composite with HT. Room-temperature tensile test results showed that ultimate tensile strength (UTS), yield strength (YS), and elongation (El) of ECAPed composites with HT were 16.1%, 23%, and 27.3% larger than that without HT, respectively.

## 1. Introduction

Due to high strength, corrosion resistance at room temperature, Mg-9%Al-1%Zn (AZ91) has been used as the most common and cost-effective commercial Mg alloy [1,2]. Unfortunately, massive secondary phases of Mg_17_Al_12_ in the form of coarse network structure deteriorate the strength at elevated temperature [3,4]. It has been reported that Si alloying in Mg-Al based alloys could improve the thermal stability; however, the existence of Chinese script-shaped Mg_2_Si decreases the mechanical properties of Mg-Al-Si materials [5]. Recently, it has been reported that SiC nanoparticles (n-SiCp) addition [6] could refine the grain size of matrix in the SiCp/AZ91 nanocomposite and modify the morphology of Mg_17_Al_12_ phase from coarse plates to lamellar precipitates, resulting in simultaneous enhancement of strength and ductility. However, it is difficult to add nanoparticles into the matrix alloy and achieve dispersed distribution due to its large specific surface area. Semisolid stirring-assisted ultrasonic vibration [6] was used to fabricate a sample of as-cast n-SiCp/Mg-Al-Si composite. Although the mechanical properties were improved to an extent, it is inevitable that blowholes and mixtures were produced during the stirring process. To obtain refined microstructures and further improve mechanical properties, several conventional deformation processes (e.g., extrusion, rolling, and forging [7,8,9]) have been applied to n-SiCp-reinforced magnesium matrix composite. Previous studies [5,10] have shown that equal channel angular pressing (ECAP) could refine the grains of n-SiCp/Mg-Al-Si composite and modify the morphology and distribution of the secondary phase Mg_17_Al_12_ and Mg_2_Si in n-SiCp/Mg-Al-Si composite effectively. However, the network Mg_17_Al_12_ cannot be distributed uniformly when the as-cast n-SiCp/Mg-Al-Si composites are subject to ECAP [5]. It is necessary to find a method to eliminate the amount of coarse pre-existing Mg_17_Al_12_ phase. The homogenization treatment was applied on the AZ91 by dissolving the eutectic Mg_17_Al_12_ second phases and homogenizing solute atoms in the Mg matrix to reduce the potential sites of intercrystalline cracking and improve the workability of AZ91 [11].

According to previous reports, the homogenized and compressed composite exhibited much higher ultimate tensile strength than that of the compressed alloy without homogenization [12,13]. Similarly, Yuan et al. [14] reported that improved strength and ductility of ZK60 can be achieved by homogenization treatment prior to ECAP, which was attributed to the activated dynamic precipitation of MgZn_2_ during the severe plastic deformation (SPD). 

Even though a great deal of work has been done, the influence of initial grain size, morphology, and amount of reinforced second phase on the microstructural evolution and tensile properties of semi-solid casted Mg-9Al-1Si-1SiC composite fabricated by assisted ultrasonic vibration and processed by ECAP still need further investigation. Thus, the microstructural evolution and resultant tensile properties of Mg-9Al-1Si-1SiC composites subjected to different pre-treatments were studied in detail.

## 2. Experimental Procedures

### 2.1. Materials and ECAP Experiment

Mg-9Al-1Si alloy was melted in a crucible under a protective atmosphere of CO_2_ and SF_6_. Pure Mg, Al, and Al-30% Si (wt %) master alloy were added to the melt. n-SiCp with an average size of 60 nm was used as reinforcement. The n-SiCp was pre-heated to 550 °C before adding to the melt. The Al-Si master alloy was added to the melt at 720 °C and held for 20 min, and then cooled down to 590 °C. Then, 1 wt % preheated SiC nanoparticles were quickly added into the semisolid slurry and stirred for 15 min. After that, the temperature was adjusted to 690 °C to make sure that the slurry converted into liquid state. During the process, the stirring rate was adjusted to 100 r/min. When the temperature reached 690 °C, the stirring process was stopped. At the same time, the ultrasonic probe was placed over the melt in the crucible to preheat for 15 min and then was dipped into the melt for ultrasonic treatment for 20 min. The power and frequency of the ultrasonic treatment device were 1.6 kW and 20 kHz, respectively. The purpose of ultrasonic treatment was to make the n-SiCp disperse uniformly in the matrix. When the ultrasonic process was stopped, the temperature was elevated to pouring temperature of 720 °C and held for 30 min. Then, n-SiCp/Mg-9Al-1Si magnesium matrix composite melt was poured into a preheated mold with a diameter of 40 mm and length of 115 mm. The samples were machined into 12 mm × 12 mm × 55 mm to fit the die used in the ECAP process. Homogenization treatment (HT) was carried at 420 °C for 24 h and then cooled to room temperature in warm water before ECAP. 

**Figure 1:**
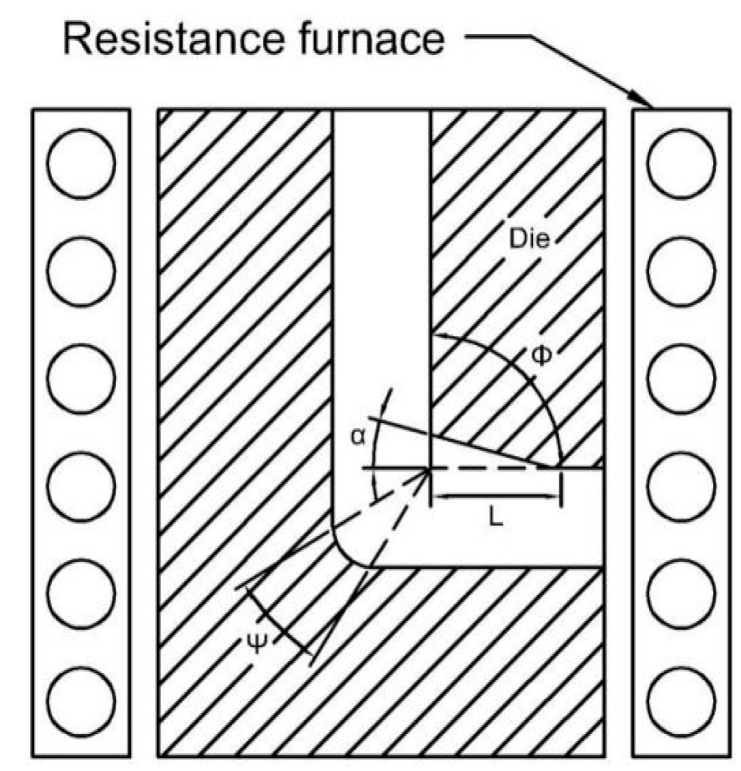
Schematic illustration of equal channel angular pressing (ECAP).

A die with internal angle, Φ, of 90° between the two equal channels and the external curvature of the point of intersection of the two channels (Ψ) of 16° was designed for the ECAP process. The ECAP experiment was conducted at 360 °C for four passes. Route Bc [15,16,17] was selected and pressing speed was 2.0 mm/min. Figure 1 shows the schematic illustration of ECAP. The samples for ECAP were ground with 400, 800, and 1200 grit papers to avoid stress concentration and were scribbled with pot lead and Vaseline.

### 2.2. Microstructures and Mechanical Testing

The microstructures of the specimens were observed by a Leica 2700 M light optical microscope (LOM, Leica Microsystem GmbH, Wetzlar, Germany). The second phases of Mg_17_Al_12_ and Mg_2_Si composition were determined by a MIRA3 scanning electron microscope (SEM, TESCAN Ltd., Brno-Kohoutovice, Czech Republic) equipped with an energy dispersive spectrometer (EDS). The existence of Mg_17_Al_12_ phase was certified by a JEM-2100F transmission electron microscope (TEM, JEOL Ltd., Tokyo, Japan). The average grain size, the amounts of both dynamically recrystallized (DRXed) grains and precipitates were calculated from the number and/or area fraction using at least ten micrographs by Image-Pro Plus 6.0 software (Media Cybernetics, Rockville, MD, USA). (0002) pole figures of the ECAPed samples were performed with a Y-2000 X-ray diffractometer (XRD, Cu-Ka, Dandong Ray instrument Co., Ltd., Dandong, China). Samples for microstructure analysis were prepared by the conventional mechanical polishing and etching using 4.2 g picric acid, 10 mL acetic acid, 90 mL alcohol, and 10 mL distilled water. Specimens for TEM observation were prepared by grinding–polishing to produce a foil of 30 µm thickness followed by punching 3 mm diameter disks. The disks were ion beam-thinned. The tensile test was conducted in a WDW-100kN tensile machine (Jinan Test Machine Co., Ltd, Jinan, China) at room temperature, and the velocity was controlled at 0.5 mm/min. Before conducting the tensile test, the prepared samples were ground with sand paper. 

## 3. Results and Discussions

### 3.1. Microstructures

**Figure 2:**
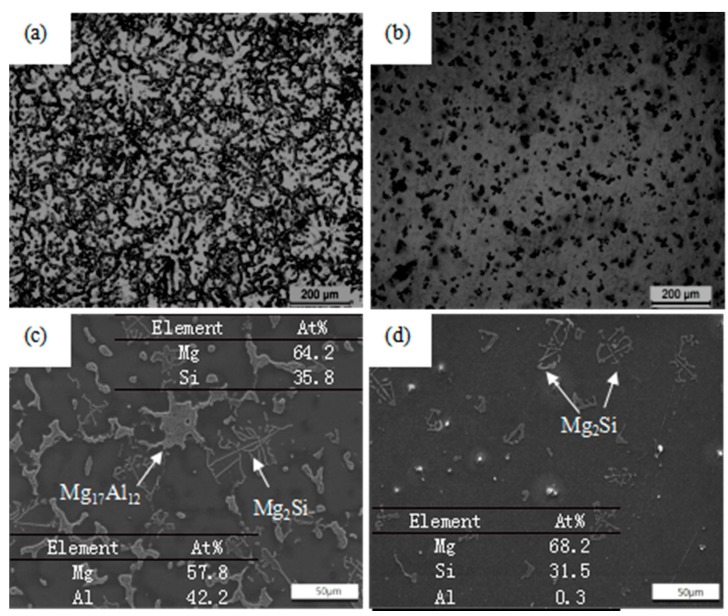
Optical, SEM (scanning electron microscope) micrographs, and EDS (energy dispersive spectrometer) results of Mg-9Al-1Si-1SiC composites prior to ECAP: (**a**,**c**) without HT (homogenization treatment); (**b**,**d**) with HT.

In Figure 2a,c, it is apparent that as-cast Mg-9Al-1Si-1SiC composite exhibits a typical dendritic microstructure with primary α-Mg matrix, secondary γ-phase (Mg_17_Al_12_), and Chinese script-shaped phases (Mg_2_Si). In addition, EDS results of secondary phases are also given in Figure 2c. It can be seen from Figure 2b,d that most of the γ-phases disappear and all the Mg_2_Si phases remain after HT. According to the Mg-Al binary phase diagram, the Mg_17_Al_12_ phase has a melting point of 458 °C and the equilibrium solid solubility of Al in Mg at 420 °C is approximately 12 wt %, so few Mg_17_Al_12_ phases could remain in the composite after HT. Meanwhile, Mg_2_Si phase with a higher melting point still existed in the α-Mg matrix. The different EDS results in Figure 2c,d illustrate that the disappearing secondary phases are Mg_17_Al_12_, which is in agreement with the facts. Generally, the changes of the amount of second phases have a significant effect on the various ECAP morphologies, as well as related tensile properties, which will be discussed in the following sections.

**Figure 3:**
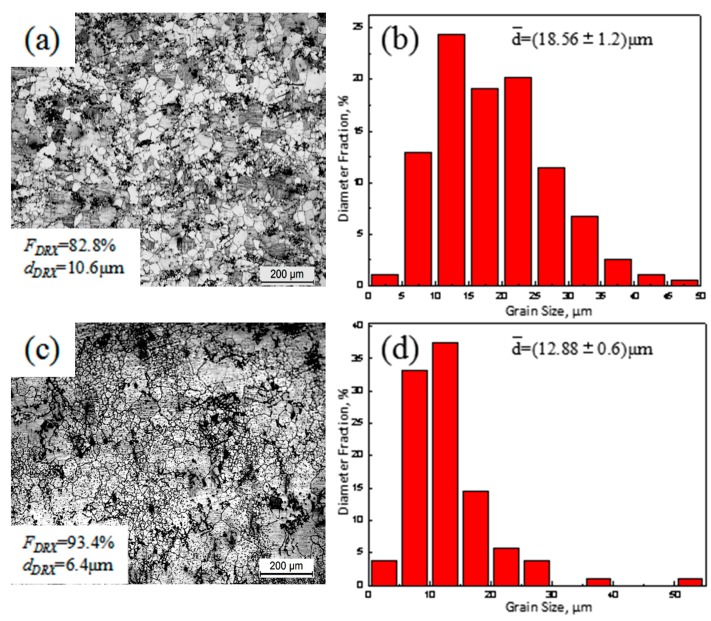
Optical micrographs and grain size distribution of ECAPed Mg-9Al-1Si-1SiC composites: (**a**,**b**) without HT; (**c**,**d**) with HT.

A microstructure with nearly equiaxed coarse grains is observed in Figure 3a. In addition, the measured area fraction and average size of DRXed grains (*F_DRX_* and *d_DRX_*) for ECAPed Mg-9Al-1Si-1SiC composite without HT were ~82.8% and ~10.6 μm (as shown in Figure 3a), respectively. The grain size distribution is given in Figure 3b, and the average grain size of ECAPed Mg-9Al-1Si-1SiC composite without HT was 18.56 μm. It can be seen from Figure 3c that ECAPed Mg-9Al-1Si-1SiC composites with HT exhibited a typical bimodal structure, which consisted of fine and equiaxial DRXed grains and coarse un-recrystallized grains. A previous study [18] reported that a bimodal structure is beneficial to the simultaneous improvement of the strength and ductility. Similarly, the measured area fraction and average size of DRXed grains (*F_DRX_* and *d_DRX_*) for ECAPed Mg-9Al-1Si-1SiC composite with HT were ~93.4% and ~6.4 μm (as shown in Figure 3c). As indicated, the measured area fraction (*F_DRX_*) was 12.8% larger and the average size of DRXed grains (*d_DRX_*) for ECAPed Mg-9Al-1Si-1SiC composite with HT was 39.6% smaller than that without HT. The results above point to a decrease of average grain size in ECAPed Mg-9Al-1Si-1SiC composite with HT. The grain size distribution is exhibited in Figure 3d, and the average grain size of ECAPed Mg-9Al-1Si-1SiC composite with HT was 30.6% smaller than that without HT. This can be explained by the production of numerous precipitates in ECAPed Mg-9Al-1Si-1SiC composite with HT during ECAP process, which hinders the DRXed grains’ growth by pining grain boundary.

**Figure 4:**
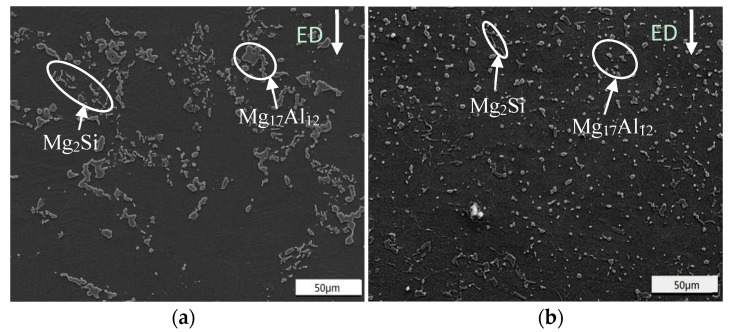
SEM micrographs of ECAPed Mg-9Al-1Si-1SiC composites: (**a**) without HT; (**b**) with HT. ED: extrusion direction.

Figure 4 displays the SEM micrographs of ECAPed Mg-9Al-1Si-1SiC composites without and with HT, respectively. After ECAP, Mg_17_Al_12_ phase and Mg_2_Si were both segregated into fragments (as shown in Figure 4a). The obvious agglomeration is also observed in Figure 4a, and the sizes of most Mg_17_Al_12_ particles are above 10 µm. It can be seen from Figure 4b that both Mg_17_Al_12_ precipitates and Mg_2_Si phases were rearranged and uniformly distributed. In addition, the morphology of the initial Chinese script Mg_2_Si changed into a needle-like morphology. Meanwhile, finer Mg_17_Al_12_ phases with size of smaller than 10 µm precipitated through dynamic precipitation [10] in ECAPed Mg-9Al-1Si-1SiC composite with HT. The results revels that HT contributes to a more refined and uniform microstructure.

**Figure 5:**
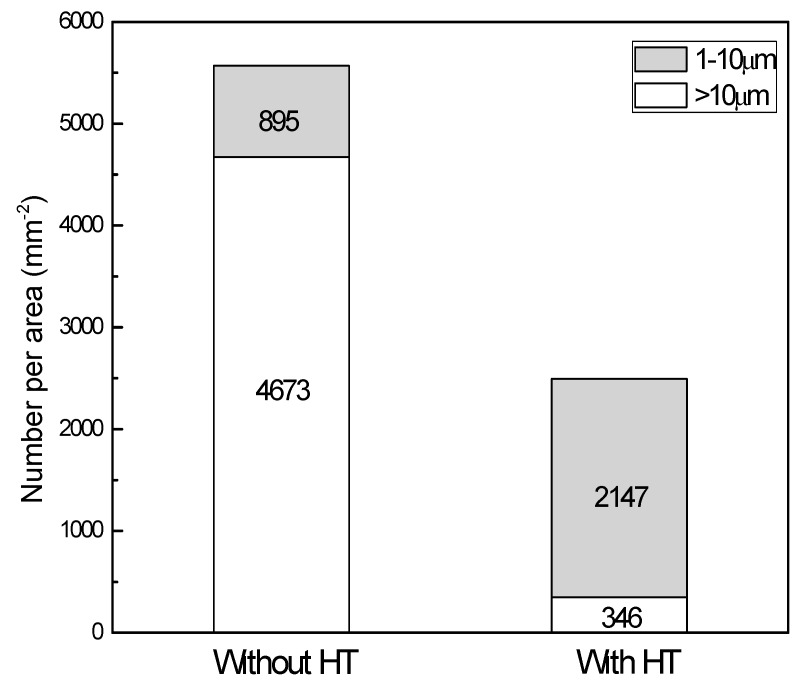
Particle-size distribution and number per area for ECAPed Mg-9Al-1Si-1SiC composites.

The number per area of Mg_17_Al_12_ particles was larger than 1 µm in the ECAPed Mg-9Al-1Si-1SiC composite, as shown in Figure 5. As indicated, the number per area of particles ranging from 1 µm to 10 µm in size of the ECAPed composite with HT was larger than that of their counterpart without HT. It is well known that particles with size 1~10 µm can act as nucleation sites for DRX during hot deformation, because of the higher dislocation density and large orientation gradient induced at the deformed zones in the vicinity of the particles [19]. This phenomenon is known as particle-stimulated nucleation (PSN), and has been widely observed in wrought Mg alloys [20,21]. For this reason, a larger amount of particles with size of 1~10 µm led to a higher fraction of DRXed grains in ECAPed Mg-9Al-1Si-1SiC composites with HT.

Furthermore, the existence of SiC nanoparticles has a positive effect on the uniform distribution of fine Mg_17_Al_12_ and Mg_2_Si particles by pinning effect [22,23]. Therefore, the grains of ECAPed composite with HT were greatly refined.

**Figure 6:**
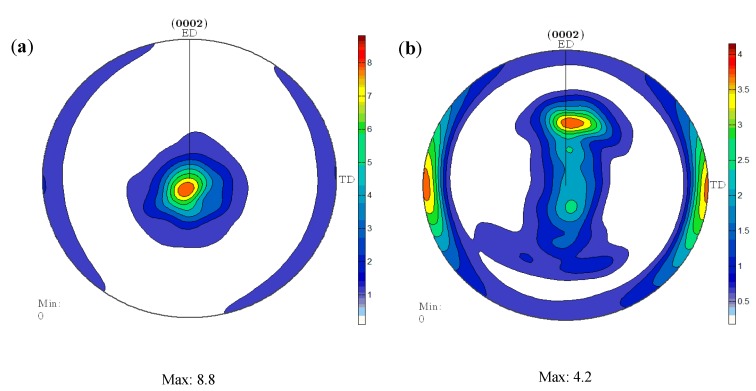
The (0002) pole figures of ECAPed Mg-9Al-1Si-1SiC composites: (**a**) without HT; (**b**) with HT.

Figure 6 shows the (0002) pole figures of ECAPed Mg-9Al-1Si-1SiC composite without HT and with HT, respectively. As indicated, the intensity of the basal texture of ECAPed Mg-9Al-1Si-1SiC composite without HT and with HT were 8.8 and 4.2, respectively. The c-axis of most grains rotated approximately 90° with respect to the extrusion direction (ED). However, some other grains tilted 30° with respect to the extrusion direction. Furthermore, a previous study [21] indicated the DRX grains form from the randomly oriented grains. Thus, a larger DRX fraction will increase the randomness of the grain orientation and finally decrease the texture intensity [24]. Besides, Mg_17_Al_12_ phases precipitated during ECAP provide more randomly oriented nuclei, and contribute to blocking the mobility of dislocations and grain boundaries. In other words, the larger the amount of dynamic precipitates, the weaker the texture of the composite. The aforementioned texture characteristics in ECAPed samples with HT will be beneficial to ductility.

### 3.2. Mechanical Properties

**Figure 7:**
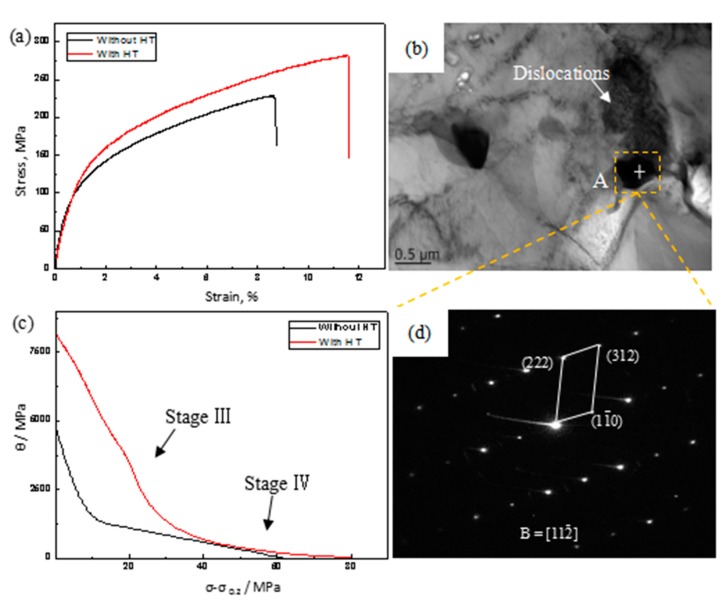
The tensile properties and bright-field TEM image of ECAPed Mg-9Al-1Si-1SiC composites: (**a**) typical tensile stress–strain curves; (**b**) dislocations; (**c**) work hardening curves; (**d**) selected area diffraction patterns of point A.

Figure 7a shows the tensile stress–strain curves of ECAPed Mg-9Al-1Si-1SiC composites without and with HT. The results show that ultimate tensile strength (UTS), yield strength (YS), and elongation (El) of the ECAPed composite with HT were 16.1%, 23%, and 27.3% larger than that of ECAPed composites without HT, respectively. The effect of HT on the mechanical properties of the ECAPed composites is remarkable. This could be attributed to the greatly refined matrix grain size and a more uniform distribution of second phases [25]. It is generally believed that the YS of wrought magnesium alloys can be associated with grain size, dynamic precipitation, and texture intensity. The YS value for metals and alloys is calculated by Hall–Petch relation [26]:

(1) σy=σ0+Kd−12

where $\sigma_{y}$ is the YS, $\sigma_{0}$ is the material constant, and *K* is the Hall–Petch slope, with a value of 300 MPa μm^1/2^. Increment in YS by grain refinement from 10.6 μm to 6.4 μm was about 26.4 MPa, suggesting that a decrease of DRXed grain size could partially enhance the strengthening effect. In addition, the precipitated Mg_17_Al_12_ particles during ECAP can serve as obstacles to dislocation movement based on Orowan mechanism [27,28]. In general, the precipitated small particles act as obstacles for the grain boundary migration and greatly hinder the growth of matrix grains, which results in fine grain size in the ECAPed Mg-9Al-1Si-1SiC composite with HT and make a great contribution to grain boundary strengthening. Similarly, the dislocations piled up around second phase precipitates in the ECAPed Mg-9Al-1Si-1SiC composite with HT, as observed in Figure 7b. The electron diffraction patterns of point A (as shown in Figure 7d) indicate that the phase is Mg_17_Al_12_. Thus, it can be seen that Orowan mechanism plays an important role in the improvement of mechanical properties of ECAPed Mg9Al-1Si-1SiC composites.

It is noteworthy that ECAPed Mg-9Al-1Si-1SiC composite with HT exhibited better ductility than the one without HT. Previous study has revealed that a more random texture in the extruded alloy with T4 could lead to more dislocation slip, giving rise to an increment of El [29]. Work hardening behavior of ECAPed alloys can be described by work hardening rate: θ=∂σ/∂ε, where σ and ε are the true stress and true strain of the alloy, respectively. The $\sigma_{0.2}$ is subtracted from σ, and thus $\sigma -\sigma_{0.2}$ is related to the dislocation contribution to the flow stress. Figure 7c shows the work hardening rate (θ) versus net flow stress $\sigma -\sigma_{0.2}$ of ECAPed Mg-9Al-1Si-1SiC composites without and with HT. It is clearly observed that there is no initially linear hardening behavior (stage II), only a dynamic recovery stage (stage III) and a large-strain work hardening stage (stage IV) appear in the θ – ($\sigma -\sigma_{0.2}$) curves of the present alloys [21,30,31]. Generally, stage II is related to high dislocation density. However, high dislocation density is attributed to ultrafine DRXed grains and dispersed dynamic precipitates with nanoscale size, which are not observed in our study. Therefore, stage II does not occur. Meanwhile, it can be seen from Figure 7c that the work hardening rates of ECAPed Mg-9Al-1Si-1SiC composite without and with HT demonstrated an almost linear decrease with increasing stress in stage III, but the ECAPed Mg-9Al-1Si-1SiC composite without HT dropped faster. It has been proved that the decreasing grain size is beneficial for dislocation slipping and increasing dynamic recovery rate [32]. Since the average grain size of ECAPed Mg-9Al-1Si-1SiC composite with HT is small, it can be inferred that such fine grains play an important role in the decrease of θ value at stage III. Stage IV is the large-strain work hardening stage, which refers to the formation of a dislocation cell structure [33]. In addition, the work-hardening exponents obtained from the Hollomon equation ($\sigma =K\varepsilon^{n}$*,* where *K* is the strength coefficient) were calculated as 0.56 and 0.78 for ECAPed Mg-9Al-1Si-1SiC composites without and with HT, respectively. It is generally known that a large work-hardening exponent leads to a low sensitivity to strain localization, resulting in enhanced elongation.

### 3.3. Fracture Surface

**Figure 8:**
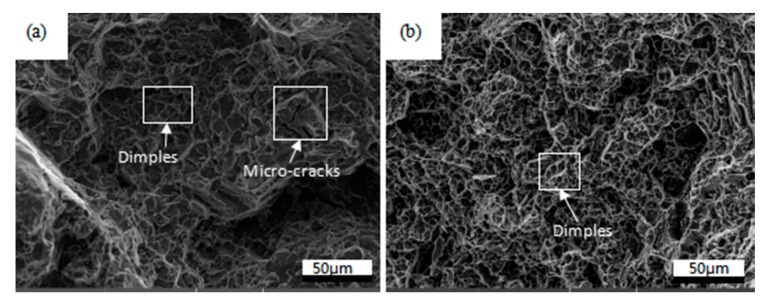
SEM fractography of ECAPed Mg-9Al-1Si-1SiC composites: (**a**) without HT; (**b**) with HT.

Figure 8 shows the fracture surfaces of samples prepared from ECAPed composites without and with HT after tensile tests, respectively. Micro-cracks and dimples can be observed in Figure 8a. Dimples are also observed in Figure 8b, but the dimples are deeper than that of ECAPed samples without HT. The micro-cracks in ECAPed Mg-9Al-1Si-1SiC composite without HT explain its poor ductility. The deeper dimples and tear ridges in ECAPed Mg-9Al-1Si-1SiC composite with HT result in its better El.

## 4. Conclusions

(1) Homogenization treatment prior to ECAP induces a more homogeneous and refined microstructure in terms of grain and second phases in the matrix. In addition, the texture intensity of ECAPed Mg-9Al-1Si-1SiC composite with HT decreases greatly due to the higher fraction of DRXed grains and second phase particles. 

(2) The UTS, YS, and El of ECAPed composites with HT were 16.1%, 23%, and 27.3% larger than that of ECAPed composites without HT, respectively.

(3) The enhanced tensile strength and ductility in ECAPed Mg-9Al-1Si-1SiC composite with HT is attributed to the combined effects of grain boundary strengthening, precipitation strengthening and texture modification.
